# Let’s DENSE: a novel protocol for efficiently collecting dense and diverse data for tactile slip detection in robotic grasping

**DOI:** 10.1038/s44182-025-00055-y

**Published:** 2025-10-13

**Authors:** Rodrigo Zenha, Brice Denoun, Andrea Cavallaro, Alexandre Bernardino, Lorenzo Jamone

**Affiliations:** 1 ARQ, EECS, Queen Mary University of London, London, UK; 2 Humanoid, London, UK; 3 7 Sensing Software, Paris, France; 4 Idiap Research Institute and EPFL, Martigny, Switzerland; 5 ISR, Instituto Superior Técnico, Universidade de Lisboa, Lisbon, Portugal; 6 Department of Computer Science, University College London, London, UK

**Keywords:** Electrical and electronic engineering, Electrical and electronic engineering

## Abstract

There is a growing interest in leveraging tactile sensing and data-driven models to enable robust robotic grasping; in this context, detecting object slip is a fundamental skill. However, the large variability in gripper-object interactions (e.g. different grasp poses, area of contact with the sensor, and directions of slip) makes the collection of suitable data to train models costly in time and resources, and current data collection protocols are oversimplified to several repetitions on a small subset of gripper-object interactions. To address this challenge, we propose DENSE, an efficient and highly reproducible protocol which is designed to capture this large variability by exploring gripper-object interactions across the object surface, and which automatically embeds straightforward labelling. We show experimentally that, compared to baseline methods, the DENSE protocol can reduce time effort by up to 50%, and models trained with the collected data improve up to 85% in their generalisation to unseen gripper-object interactions.

## Introduction

Handling arbitrary objects in unstructured environments is an open challenge for autonomous robots^1^. While vision provides meaningful information to generate a motion plan for grasping^2^, tactile sensing helps maintaining accurate information on the contact interaction between the gripper and the object^3^. For autonomous grasping, tactile sensing can enable robots to cope with uncertainties. Currently, most state-of-the-art grasp planning algorithms work in an open-loop manner^4^; i.e. after a grasp is generated, the robot executes it without modifying its behaviour, even when the object slips from the gripper. For this reason, we are interested in detecting slips that occur right after a grasp when the robot lifts the object.

As autonomous robots now have the ability to grasp a wide range of objects^2^, we argue that slip detection models should also be capable of coping with the same object variability range. First, the robot should detect slips regardless of an object’s properties, such as geometry, weight distribution or texture^5^. Second, due to real-world uncertainties, the robot should detect slips regardless of the pose of the fingertips with respect to the object^6^. In fact, small errors in perception and robot control can lead to a variety of grasp configurations, even without the whole sensor being in contact with the object^7^. Although previous works used learning techniques, including Deep Learning (DL), to detect slips on two-fingered grippers with some degree of success, the formulation of the problem and methodology to collect and label data do not account for the above variability^5,8–10^. In addition, collecting and labelling large amounts of tactile data in less constrained scenarios (e.g. autonomous grasping) is very challenging and greatly impacts the performance of classifiers^11^.

Robotic slip detection requires two components: a sensor that captures signals related to the physical interaction between a gripper and an object (e.g. vibrations, region of contact, distributed forces)^12,13^, and a classifier (a model) identifying if such data corresponds to a slip event ^8^. Recent works have proposed data-driven approaches, which require collecting and labelling tactile data for both slip and static contact events between objects and a tactile sensor to train the slip classifier^14–18^.

A Support Vector Machine (SVM)^19^ can discriminate gross slip events^14^ based on processed data captured with the TacTip sensor^20^. Although the SVM is trained with data collected on five objects only, the fitted model generalises well to six new objects. Similarly, Random Forests (RF)^21^ applied to the Fast Fourier Transform^22^ of the raw data can classify object slips with 80% accuracy^18^.

Methods that rely on DL to learn features^23^ from raw or calibrated data ^15,16,24^ avoid the need for data processing before classification. In ^15^, a Convolutional Long Short-Term Memory is trained from the raw data of a BioTac sensor^25^ to classify slips (and slip direction). Although the model can generalise to different textures and slip velocities, the method requires collecting a large amount of data (~85k tactile samples) and is, in practice, limited to a relatively small set of objects. This is an underlying limitation of DL models that, despite better classification performance, usually require more data to learn a given task compared to more traditional Machine Learning (ML) techniques^26,27^. This shortcoming becomes even more important when the task requires neural networks to account for several sources of variability^27^ since it can lead to a tedious and time-consuming data collection process on the hardware.

To collect large datasets with minimal effort, an increasing number of works leverage the recent progress in simulation^28^ to collect realistic tactile data^29,30^. Several works demonstrate how some tasks that rely on tactile data can be learned entirely in simulation and then be deployed on a physical robot with minimum adaptation^31–33^. However, these approaches require the underlying sensing mechanism to be simulable, which is currently mostly restricted to optical sensing, i.e. tactile sensors embedding a camera^34,35^.

As a result, training models for slip detection for non-vision-based tactile sensors requires an exhaustive data collection and labelling process. To address the challenges associated with the labelling of slip events - characterised by short-lived and hard-to-isolate phenomena—some previous work has resorted to weak labelling techniques^24^, i.e. carefully designing the grasping and data collection protocol so that all samples recorded for each experiment can be assigned one particular label. This process can result in noisy datasets—possibly mitigated by regulating the data recording time and grasp forces. However, alternative automated labelling processes (e.g., resorting to external vision^14^ or accelerometers^36^), although more accurate, are also more expensive and not easily deployed in scenarios involving robotic motion.

The data collection process can be simplified in several ways. For instance, authors tend to strategically place objects against the sensor to maximise the area of contact^9,37,38^, which is not representative of how robots can grasp objects in unconstrained environments. Other works only consider data when the robot is already holding objects in the air^14,36,39^ or is pushing them against a vertical support^9,38^. By keeping the gripper static, the variability of the process is significantly simplified since object-gripper physical dynamics, such as arm vibrations, stretches of the sensor material, object load and/or unloading, are not accounted for. To partially solve this lack of variability, James et al. have proposed inducing slips through step-wise releases of the grasp forces^14^. However, this solution is not suitable for all grippers as, for instance, cheaper grippers do not generally provide the fine finger-position control required to induce slips in a controlled manner. Similarly, other works have proposed diversifying the contacts between the gripper and objects by collecting tactile data for different object poses^24^. However, the authors mention that finding the grasp poses necessary to collect the training data requires numerous empirical trials and errors, generally leading to only a few grasp poses per object^6,10,24^—usually up to four^14,24^ or six^15,39^—for which grasping experiments are repeated several times.

In our previous work^11^, we proposed to train a RF model ^21^ based on data collected in an automated (vision-based) pick-and-place task, including object lifting. However, the model did not generalise well to new grasp attempts or new object poses. We attributed these limitations to two main reasons. First, accurately labelling each tactile sample corresponding to slip events during the motion of the robotic arm is challenging. In addition, collecting data in the wild hinders the control of the distribution and nature of slips (e.g. intensity, position), leading to the trained models being skewed to specific object-gripper interactions, thus impeding generalisation^40^.

To address these limitations, in this work, we propose the DENSE (Diverse Exploration of Natural Slip Events) protocol for collecting tactile data to train slip detection models. Unlike previous data collection approaches, grasp positions are generated according to the geometry of both the sensor and the objects of interest, which (i) does not require any prior experiments before starting to collect data and (ii) makes the process more repeatable. Moreover, this strategy allows us to capture naturally occurring slips (from less stable grasps) instead of artificially inducing them with step-wise releases of the grasp forces or human intervention^39^, thus making the method suitable for a wider generality of grippers. In addition, using a uSkin sensor^41^, we demonstrate that with fewer grasp experiments than standard data collection processes, the DENSE protocol allows us to collect more variability in the training data, improving the generalisation performance of three popular classification methods when provided with new grasp data related to new objects and new object poses.

**Fig. 1:**
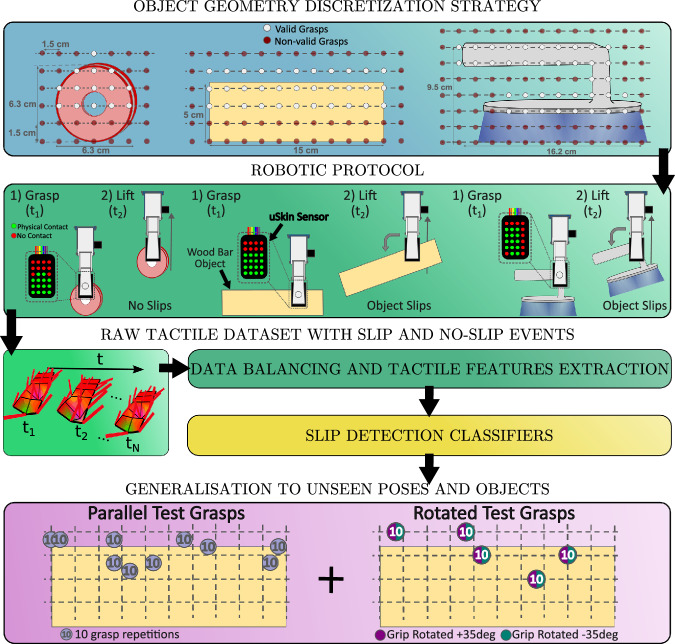
Schematic view of the proposed strategy for data-efficient robotic slip detection based on tactile information. Grasp poses are generated based on the object and the tactile sensor geometric properties. A robotic routine composed of grasping and lifting is repeated for each grasp pose, resulting in either *Slip* or *No-slip* events. The labelled tactile data is then used to train slip-detection classifiers. Finally, we evaluate the classifiers' ability to generalise to new grasp poses and objects.
- we create and share a new tactile dataset (Dense-dataset) collected with the proposed DENSE protocol, and show that our data captures more variability than data collected with state-of-the-art approaches.
- we evaluate the generalisation performance of several slip detection models trained with our Dense-dataset, and show their robustness to new objects and grasp poses.

The main contributions of this paper are summarised as follows:

- we propose DENSE, a new object-agnostic protocol to collect tactile data for training robust slip detection models (Fig. 1), which relies on few simple robot actions and a fast labelling procedure;

## Results

### The DENSE protocol

**Fig. 2:**
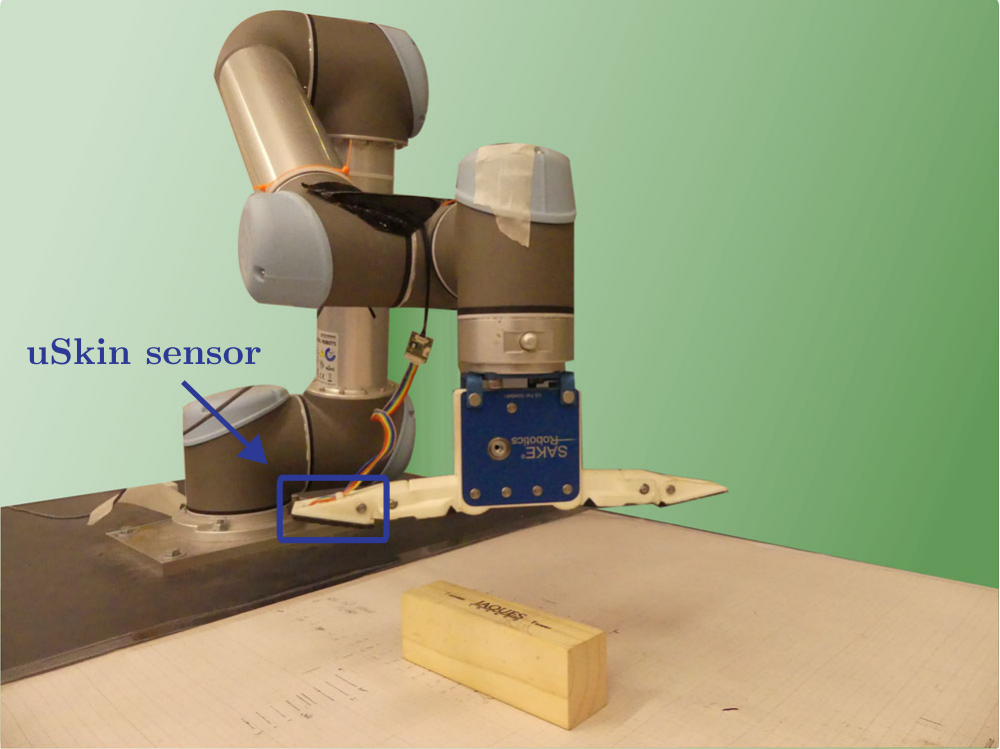
Experimental robotic setup for the tactile data acquisition during each object pick-and-lift. The uSkin tactile sensor is installed on an EZGripper two-fingered gripper, which is attached to a UR5 robot arm.

The DENSE protocol is split into three main stages, described in the next three subsections: generation of a valid set of grasp poses for each object; robotic object grasp execution and tactile data collection; and data labelling. Finally, the last subsection describes the set of objects used to build training tactile datasets. In this work, the grasps generated by the proposed protocol are performed using the EZGripper (see Fig. 2), a low-cost and underactuated dual-fingered robotic gripper. To collect tactile data, a uSkin tactile sensor, based on magnetic technology^42^, is installed on a single finger, while the other is covered with the same fabric layer as the sensor, but without any sensing elements. As a result, it is slightly more rigid, but retains the same texture, and therefore has the same (or very similar) friction coefficient.

#### Grasp pose sampling

Since grasp configurations executed by autonomous systems can result in a wide range of contact points between the fingertips of the robot and the object, we believe that the training dataset should be composed of different gripper-object interactions. For instance, the position of the sensor with respect to the object centre of mass will dictate the intensity (i.e. direction of rotation or translation, and velocity) of the slip. This is especially true when the fingertips are only partially contacting objects. To generate such variability in a repeatable and controlled manner, we propose to discretise the object dimensions with a resolution equal to half the smallest side of the bounding box of the tactile sensor, *d*, which will be referred to as the object discretisation step. The discretisation step was selected so that, during data collection, the sensor is in contact with the entirety of the (reachable) object surface at least twice across all generated grasp poses. Reducing the discretisation step would result in more grasp experiments, and therefore, more time to collect the data, while increasing it would result in the generated data not containing grasp information on some parts of the objects.

**Fig. 3:**
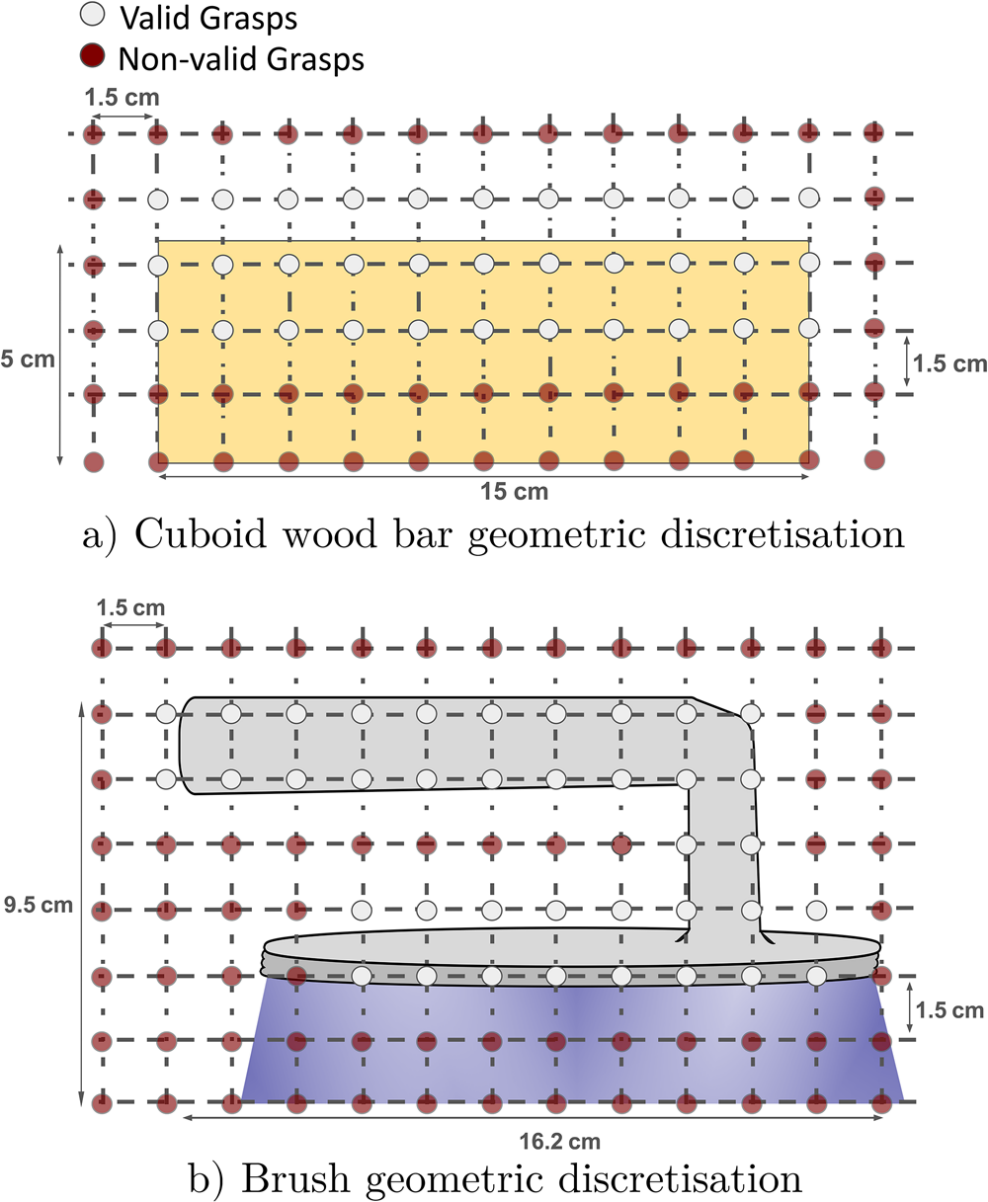
The valid and discarded grasp poses (as described in II-A) are represented as white and red circles, respectively. **a** The resulting grasp discretisation of a cuboid-shapped wood block. **b** The resulting grasp discretisation of a brush.

For an arbitrary object placed on a table, the corresponding object discretisation step corresponds to defining a virtual 2-dimensional grid (see Fig. 3)—contained within a vertical plane aligned with the major axis of the object—with a spatial resolution *d*, and covering the whole space that the fingertips of the robot can reach for a given orientation of the gripper, which is assumed to be always vertically aligned with gravity (as shown in Fig. 2). As illustrated in Fig. 3, each vertex of this virtual grid corresponds to a candidate position defining the centre of contact between the fingertips and the object. To avoid collecting data that is unrepresentative of the behaviour we try to detect, grasp configurations should be kept if and only if the object remains within the fingers of the gripper when the latter closes. Similarly, all grasp configurations resulting in undesired contact between the table and the end-effector should also be discarded. If the width of a given object is not divisible by *d*, padding is applied on each side of the object to result in a discrete number of contact points along this axis. Figure 3 shows examples of the sampled grasp configuration for two objects using this strategy, considering a uSkin sensor mounted on an EZGripper (*d* = 1.5 cm).

#### Grasp execution

**Fig. 4:**
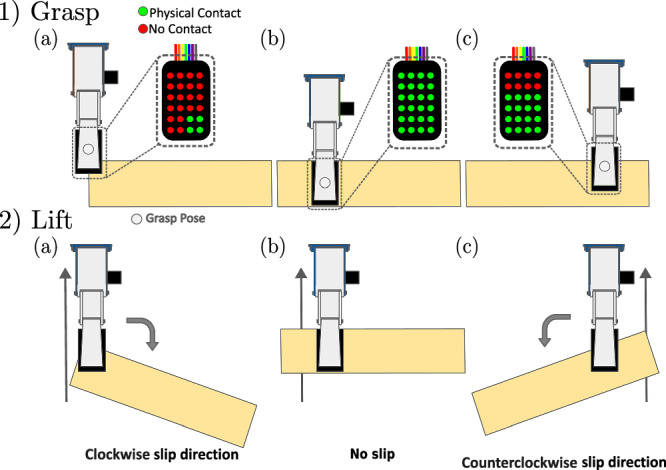
The proposed grasp execution stages 1). **a-c** Object grasp execution stage, exemplified for three different grasp poses. 2) **a-c** Object lift execution stage, illustrating the resulting stability of the same grasps.

As illustrated in Fig. 4, a grasp experiment for an isolated object with a given pose on a table consists of the following pick-and-lift procedure:

1. Move the robot arm to a given grasp configuration
2. Close the robot fingers (i.e. grasp the object)
3. Start collecting tactile data, *t*_*b*_
4. Raise the robot arm to a pre-defined pose, i.e. the robot lifts the object, lasting some period of time *T*_*r*_ (in this paper, *T*_*r*_ ≈ 0.2*s*)
5. Once the robot arm is static, wait a period of 2 s, *T*_*s*_ (*T*_*s*_ = 2*s*)
6. Stop recording tactile data, *t*_*e*_

For a given grasp pose, the above steps can be repeated *R* times. A higher number of repetitions *R* allows for recording more variability related to experimental errors (e.g. hardware controller, object placement) but requires more time to execute overall. While running each grasp experiment for the different grasp configurations, some experiments will lead to the object slipping (with rotational or translational momentum) from the gripper as soon as the robot arm moves, while others will remain firmly grasped. We believe that capturing both behaviours is crucial to train classifiers that can cope with different interactions between a gripper and a set of objects.

In practice, to simplify the experimental procedure, during the grasp execution step, instead of generating individual robot joint states corresponding to each sampled grasp of a given object, we predefine one robot joint state for each height of the grasps to be explored (e.g. three grasp heights for the cuboid wood bar object shown in Fig. 3 a)). Furthermore, graph paper is attached to the table top, so for each grasp height, the object is moved by a step of *d* cm horizontally (along the major axis of each object) until its pose matches the sampled robot grasp configuration, allowing for efficient data collection with minimal overhead. We implemented this data collection pipeline using the Grasping Robot Integration and Prototyping (GRIP) software framework^43^.

Another important factor to consider during data collection is the grasp force applied to each object. The force that can be applied varies between grippers, and it also depends on the object properties (e.g. stiffness). This work assumes rigid objects, and the grasp force to be the same for all objects and to be kept constant during the data collection procedure. The grasp force was chosen with the following criteria: large enough so that grasp poses near to the objects’ centre of mass would generally lead to stable grasps (non-slips); small enough so that some of the grasp poses would generate slips; small enough not to damage any of the objects. Based on these criteria, the chosen grasp force was approximately 10 N, which is within the sensing range of the tactile sensor (0–14 N, as reported by the manufacturer).

#### Data labelling

**Fig. 5:**
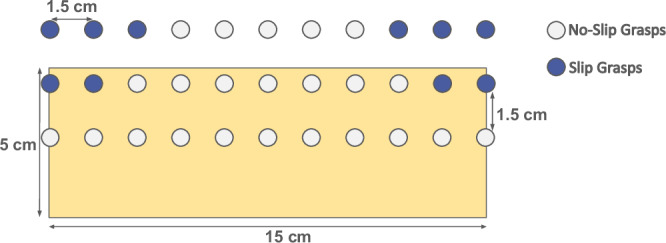
Generated grasp configurations for the wood bar, containing *Slip* events (in blue) and *No-Slip* events (in white).

Since data will be collected during the execution of grasps that involve the movement of a robotic arm, automatic labelling methods of the individual tactile sample are unfeasible without somehow controlling or limiting the grasping task^11^. Instead, to label slips for each collected tactile sample, we follow an approach similar to refs. ^24,14^, i.e. assuming that all samples from a grasping experiment correspond to the same label (*Slip* or *No-Slip*). After each experiment, all individual tactile samples recorded (sampled at 100 Hz) are labelled according to whether the experimenter observed a slipping or a stable grasp during that experiment. We believe, such an assumption to be reasonable since we record data only for 2.2 s (from the moment the object is raised above the table, *T*_*r*_ + *T*_*s*_), which does not allow objects to fall, but only to start slipping or to remain stable. If objects were to fall within the first two seconds after lifting them, we would advise experimenters to increase the grasping strength while making sure not to damage the object. In other words, we propose to rely on the observation of experimenters to label whether all samples of the sequence correspond to slips (object moves within the fingers of the robot) or a static contact. Although the resulting labels will correspond to an approximation of the real events, this approach saves labelling time and resources. Figure 5 exemplifies which grasp poses would lead to slip or no-slip events for a cuboid wood bar.

#### Data collection

In this work, to validate the effectiveness of the proposed protocol, data is collected with seven objects. These objects are an empty cardboard can, an unopened soda can, three cuboid wood bars—two of them wrapped in either baking paper or duct tape to change their respective coefficients of friction—a metal bar, and a brush. As reported in Table 1, this set of objects includes a variety of shapes (cuboid, cylindrical, composite), weights (between 47 and 356 g), dimensions and textures. The coefficient of friction of each object has been estimated by executing the experiment described in^44^ with a 500 g weight. Given the variety of object properties chosen (size, mass, and friction), it is observed that both slips and stable grasps can occur either under partial contact or full contact between the sensor and the objects, depending largely on the distance between the centre of the grasp and the centre of mass of the object.

**Table 1:** Geometrical and physical properties of the objects used for the tactile dataset collection

Object	Brush	Soda Can	Metal Bar	Carboard Can	Wood Bar	Wood Bar - paper	Wood Bar - duct tape
Object picture							
Dimensions (mm)^a^	95 × 151 × 76^**b**^	63 × 92 × 63	41 × 101 × 41	74 × 251 × 74	50 × 150 × 40	50 × 150 × 40	50 × 150 × 40
Weight (g)	165	356	159	47	124	124	124
Total Number of Grasp Poses	32	14	14	51	33	33	33
Shape	Composite	Cylindrical	Cuboid (with ridges)	Cylindrical	Cuboid	Cuboid	Cuboid
Estimated Coefficient of Friction^44^	0.061–0.224	0.082	0.102	0.163	0.204	0.245	0.306

^a^ Height × Length × Width.

^b^Brush handle width is 25 mm.

For each sampled grasp pose of each object, *R* = 10 experiments are collected and labelled, leading to a total of 2100 grasps across the seven objects. This corresponds to 70 min of tactile data, and approximately 420k tactile samples, with 230k labelled as slip events, and 190k as non-slip events. The resulting dataset has been made publicly available.

### Tactile slip detection

Previous works demonstrated the benefits of data-driven approaches to detect instances of slips from tactile data captured on a physical platform (see Section “Introduction”). To validate the effectiveness of the proposed DENSE protocol, we compare the performance of three commonly used classifiers—Random Forest ^21^, Support Vector Machine^19^ and Multilayer Perceptron^45^—to detect slip or stable grasp events from the individual tactile datapoints. Each classifier is trained with three different training sets extracted from the dataset introduced in Section “The DENSE protocol”. Specifically, we are interested in inspecting the extent to which datasets generated with fewer grasp experimental repetitions following the DENSE protocol enable us to capture more variability than the data collection processes presented in previous works (described throughout the remainder of this paper as baseline approaches).

#### Training sets

This subsection defines three training datasets—all extracted from the dataset described in Section “The DENSE protocol”—for which the variability of tactile samples will be quantified and compared. Two of these datasets correspond to the training sets that would result from collecting data following the same approach as previous works (baseline datasets). The last one is a training set collected using the DENSE protocol for *R* = 1 (Dense-dataset).

**Fig. 6:**
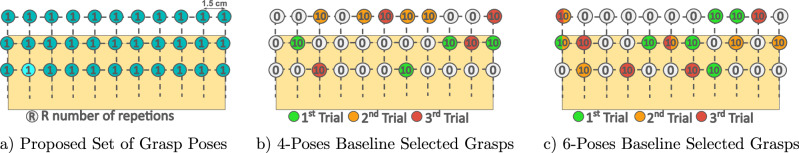
Selected set of grasp configurations for the Dense-Dataset, Baseline-4, and Baseline-6 datasets. **a** Proposed set of grasp poses that compose the Dense-Dataset. **b** Independent repetitions of the data collection process for Baseline-4. **c** Independent repetitions of the data collection process for Baseline-6. The numbers represent the number of repetitions, R, selected for each grasp configuration. Colours green, orange and red represent the 4 or 6 grasp poses selected for each independent repetition of the data collection process.

*Baseline datasets:* Typically, data collection baseline approaches involve selecting between four to six grasp poses—chosen by the experimenter through a trial and error approach^6,10,24^—for each object to generate a training dataset with an equivalent number of slip and no-slip occurrences. To better quantify the impact of varying the number of grasp poses used to train the slip models (which will be evaluated for their generalisation capabilities), we define two training sets composed of data collected from 4 or 6 grasp poses, for which *R* = 10 experiments are considered. Similarly to the protocol described in ref. ^24^, we ensure that for each object, half of the extracted poses correspond to slip events, and half of them correspond to static contact between the gripper and the object. However, we argue that, by design, this approach to defining a training set for tactile slip detection is prone to result in classifiers with varying performance depending on the extracted grasp poses. In fact, a classifier trained on 4 or 6 grasp poses spread all over the object is more likely to generalise better to new grasp poses than a classifier trained with the same number of grasp poses but located only on one side of an object. To validate this assumption, and as illustrated in Fig. 6b, c, we create, for both approaches, three training datasets for which the grasp poses resulting in slips and no-slips are randomly selected for each object. When selecting four grasp poses, the three resulting datasets will be referred to as Baseline-4.1, Baseline-4.2 and Baseline-4.3. A similar naming convention is used with datasets composed of six grasp poses. Note that for the seven objects, the resulting Baseline-4 training sets contain 280 grasp experiments (4 poses × 7 objects × 10 repetitions), while Baseline-6 training sets contain 420 grasps.

*Our Dense-dataset:* The last set is designed so that slip detection models are trained with data collected across the whole surface of each object. However, instead of using the 10 repetitions of each grasp pose (which would result in a dataset composed of 2100 grasps), we select only one repetition (*R* = 1), as illustrated in Fig. 6a for the wood bar object. This set, composed of 210 grasps (across all objects), will be referred to as Dense-Dataset. Unlike the two previous baseline sets, the number of sampled grasps differs for each object (between 14 and 51 grasps, see Table 1) but results in fewer experiments in total. As a result, for each object, the number of grasp experiments leading to slip and static events is likely to be uneven, which would lead to an unbalanced set comprising more labels of one class than another. To remediate this, we propose that, for each object, a number of samples—corresponding to the difference between the total number of slip and no-slip samples—should be randomly and evenly discarded across all grasp poses whose generated samples correspond to the modal label. Similarly to the baseline datasets, we created three versions of this dataset (Dense-Dataset.1, Dense-Dataset.2, and Dense-Dataset.3) to quantify how much re-generating a dataset using our approach (i.e. re-collecting data with a new repetition for each sampled grasp pose) can impact the performance of classifiers.

#### Variability comparison

**Fig. 7:**
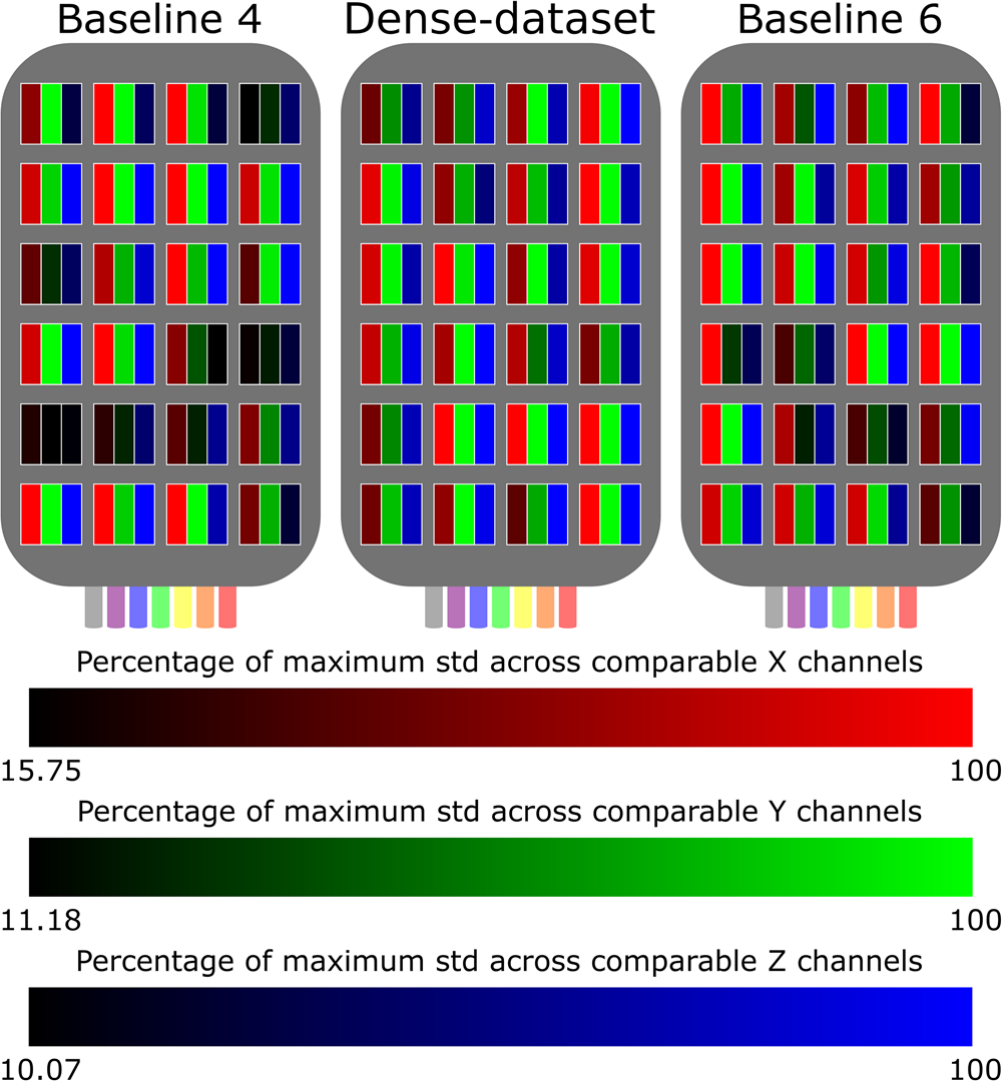
Comparison of the variability captured by each channel of the sensor (shown here in a top view) for the Baseline-4, Dense-Dataset, and Baseline-6 training sets. The lighter the colour of a channel, the closer it is to the maximum standard deviation captured by the same channel across the three training sets. It can be seen that the channels for the Dense-Dataset are, generally, of a lighter colour than the remaining datasets, meaning that it contains a more uniform distribution of high standard deviations across the sensor surface.

To quantify the variability of the tactile data embedded into each dataset (Baseline-4, Baseline-6, and our Dense-Dataset), we compute the percentage of maximum standard deviation, *p*(*c*_*i*_), across each channel *c*_*i*_, *c*_*i*_ ∈ {*x*_*i*_, *y*_*i*_, *z*_*i*_}, *i* ∈ {1, …, 24}. For instance, considering the sensor’s channel *x*_*i*_, *p*(*x*_*i*_) becomes:

$$\begin{array}{ll}p({x}_{i})=\frac{\sigma ({x}_{i})}{\alpha (x)}\times 100,i\in \{1,\ldots ,24\},\\\alpha (x)=max(\sigma ({x}_{1}),\ldots ,\sigma ({x}_{N})),N=24,\\\sigma ({x}_{i})=\sqrt{\frac{\sum {({x}_{i}-\mu ({x}_{i}))}^{2}}{b}},\end{array}$$

where *μ*(*x*_*i*_) is the mean value of all datapoints of channel *x*_*i*_ in the corresponding dataset. Figure 7 illustrates the *p*(*c*_*i*_) obtained for each channel of the sensor, for each of the training datasets described above. It can be seen that the Dense-Dataset (ours) contains a more uniform distribution of high standard deviations across the sensor surface (represented by the lighter red, blue and green colours) compared to the Baseline-4 and Baseline-6 training sets, where some channels present very low standard deviations (represented by darker colours).

#### Generalisation testing

**Fig. 8:**
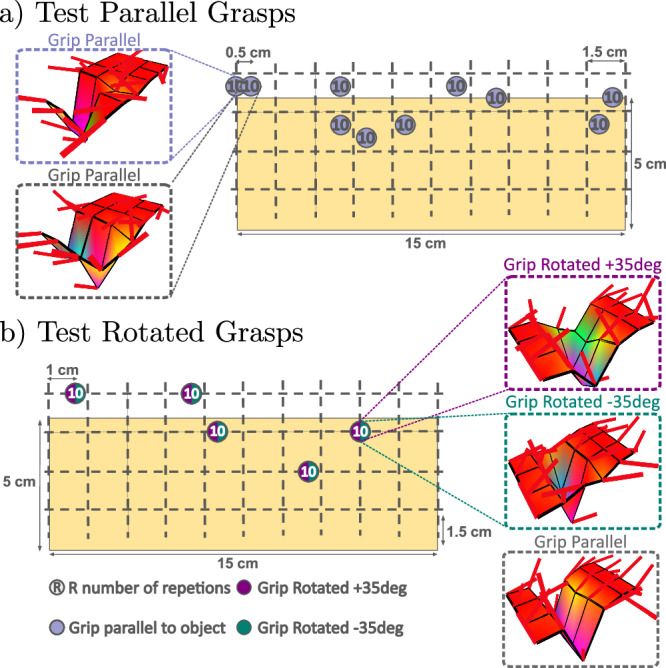
Positions of the grasp configurations used to collect the test sets related to the wood bar. **a** 10 randomly sampled positions for which the gripper keeps the same orientation as in the training data. **b** 5 grasp positions for which tactile data is collected when the gripper is rotated with a yaw angle of ±35^∘^. Examples of tactile imprints of each test set are illustrated, showing the variability observed in the data.

In order to assess the generalisation of slip detection models to new grasp poses, we collect—for all the seven objects—two additional sets of grasping experiments containing variabilities likely to be encountered in real-world scenarios. The first set of experiments consists of randomly sampling and grasping each object in 10 new positions outside of the proposed discretised space, ensuring that half of them lead to slip events. For each position, the tactile data from 10 repetitions is gathered and labelled. The resulting dataset is referred to as Parallel-Test (PT). The second set consists of grasping each object in an additional five randomly sampled positions, but in which the gripper is rotated with a yaw angle of ±35^∘^. Again, for each gripper rotation, the tactile data from *R* = 10 repetitions are gathered. This subset will be referred to as Rotated-Test (RT). Figure 8 illustrates the two additional sets of experiments collected for the wood bar. In total, 200 new grasp experiments are performed per object.

Since this study also aims at evaluating the impact of the Dense-dataset on the generalisation power of resulting ML models—which includes predicting slips of unknown objects—we propose to define four scenarios in which the number of objects *O* = {5, 4, 3, 2} used to train the classifiers vary. Since the combination of properties associated with the objects used for training is likely to drive the classifier performance, we define, for each scenario, different unique training subsets. For instance, when training classifiers with *O* = 5, we split the seven objects into five subsets, *S* = 5, of five objects each, and each subset is used to train the models independently. The performance of the models is evaluated through cross-validation across all individual subsets, using the data of the remaining two objects (of each subset) for generalisation testing. Following the same approach, when training models with *O* = 4, the group of objects is split into eight subsets, *S* = 8, since more combinations of objects and object properties are available. For *O* = 3, the objects are divided into *S* = 9, and finally, for *O* = 2, they are divided into *S* = 12. The final number of subsets *S* considered for each *O* is obtained from combining objects with a wide variety of distinct physical and geometrical properties, shown in Table 1. The generalisation power of each slip detection model trained on a given subset is therefore evaluated on three test sets:

1. Tactile data corresponding to the Parallel-Test collected for all the objects used for training (PT);
2. Tactile data corresponding to the Rotated-Test collected for all the objects used for training (RT);
3. Complete tactile dataset of novel objects, i.e. not used during training, also including their associated Parallel-Test and Rotated-Test sets.

As previously mentioned, the first two test sets are meant to evaluate the generalisation power of a model to new grasps for known objects, while the last test set aims at evaluating the generalisation power to unknown objects.

**Fig. 9:**
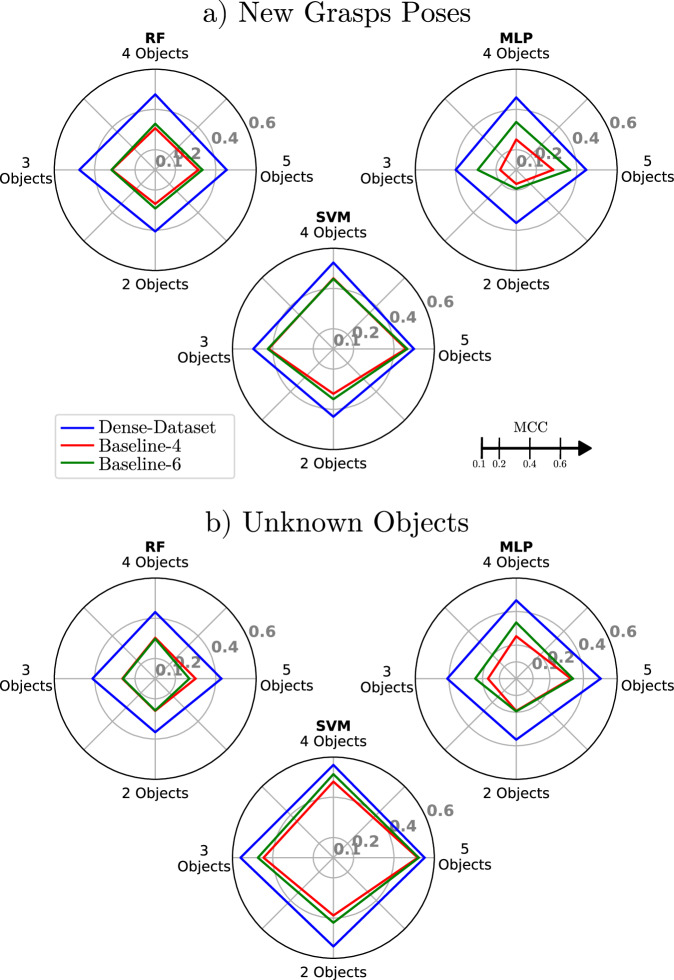
Generalisation test scores of the RF, SVM, and MLP slip classifiers when trained with data from *O* = {5, 4, 3, 2} and collected following either Baseline-4, Baseline-6 or the DENSE protocol. **a** Generalisation results to new grasp poses (PT + RT). **b** Generalisation results on unknown objects.

In summary, in this section, we described that nine datasets have been generated from three repetitions of each of the three data collection approaches to be evaluated (Fig. 6), on the objects reported in Table 1: Dense-Dataset.{1,2,3}, Baseline-4.{1,2,3} and Baseline-6.{1,2,3}. The resulting datasets captured different overall degrees of variability of the sensor-object interactions, as reported in Fig. 7. Each dataset is divided into training subsets containing a varying number of objects (between 2 and 5), which are then used to train three ML classifiers. For each subset, the remaining objects (not used in training) are used to test each classifier for generalisation to new objects. Furthermore, for all objects, new PT and RT test sets were collected (as illustrated in Fig. 8) and used to test the generalisation capabilities of the classifiers, both to objects seen during training and new objects. The results are shown in Fig. 9a, b, respectively, and will be discussed in detail next.

For each test set, the performance of the classifier is quantified using the Matthews Correlation Coefficient (MCC)^46^. The results will be reported as the average and standard deviation of the test MCC scores of each classifier computed over the three repetitions of each training set (e.g. Dense-Dataset.{1,2,3}). We believe that such results better reflect the impact of each data collection approach on the repeatability of the classification performance. Next, we will discuss the generalisation performace of the resulting ML models when tested on new grasp poses of the same objects used for their training (Tables 2 and 3), and quantify and compare the generalisation power of the same classifiers for objects (and grasp poses) not seen during training (Tables 4 and 5). An overview of the results presented is illustrated in Fig. 9, illustrating each classifier MCC generalisation test scores to new object grasp poses (including both PT and RT) and to new objects, when trained with data from *O* = {5, 4, 3, 2} collected with each of the three evaluated approaches.

**Table 2:** Generalisation performance of the three classifiers on data corresponding to new grasp poses (PT and RT datasets) collected on each subset of five objects used for training

		Random Forest						Support Vector Machine						Multi Layer Perceptron					
		Dense-Dataset		Baseline-4 (200 grasps)		Baseline-6 (300 grasps)		Dense-Dataset		Baseline-4 (200 grasps)		Baseline-6 (300 grasps)		Dense-Dataset		Baseline-4 (200 grasps)		Baseline-6 (300 grasps)	
Subset (Dense-Dataset # of grasps)		PT	RT	PT	RT	PT	RT	PT	RT	PT	RT	PT	RT	PT	RT	PT	RT	PT	RT
**1**(164)	Avr.	**0.35**	**0.45**	0.32	0.26	0.31	0.33	**0.58**	**0.45**	0.53	0.42	0.50	0.41	**0.48**	**0.35**	0.26	0.26	0.33	0.33
	std	0.01	0.02	0.03	0.02	0.02	0.1	0.03	0.02	0.02	0.02	0.05	<0.005	0.01	0.05	0.05	0.08	0.12	0.09
**2**(127)	Avr.	**0.41**	**0.46**	0.34	0.27	0.34	0.29	0.53	0.38	0.54	**0.39**	**0.54**	0.36	**0.47**	**0.36**	0.30	0.22	0.27	0.36
	std	0.01	0.01	0.09	0.06	0.05	0.17	0.01	0.02	0.03	0.06	0.01	0.03	0.04	0.05	0.21	0.07	0.11	0.13
**3**(126)	Avr.	**0.39**	**0.45**	0.33	0.35	0.33	0.38	0.38	**0.42**	0.39	0.37	**0.40**	0.40	**0.33**	**0.40**	0.19	0.29	0.27	0.39
	std	0.03	0.02	0.03	0.11	0.04	0.11	0.01	<0.005	<0.005	0.03	0.06	0.04	0.05	0.05	0.16	0.14	0.07	0.15
**4**(163)	Avr.	**0.42**	**0.59**	0.30	0.30	0.23	0.41	**0.53**	**0.56**	0.46	0.46	0.44	0.53	**0.48**	**0.51**	0.31	0.36	0.33	0.46
	std	0.01	0.03	0.04	0.03	0.14	0.19	0.01	0.01	0.07	<0.005	0.03	0.07	0.09	0.09	0.07	0.07	0.09	0.04
**5** (163)	Avr.	**0.42**	**0.56**	0.29	0.33	0.31	0.37	**0.55**	**0.56**	0.49	0.50	0.49	0.54	**0.52**	**0.53**	0.29	0.31	0.41	0.46
	std	0.01	0.01	0.07	0.03	0.11	0.16	0.01	0.03	0.04	<0.005	0.03	0.09	0.02	0.02	0.15	0.08	0.05	0.12

The average and standard deviation of MCCs are computed across three repetitions of each data collection approach. The best results obtained for each model and subset are highlighted in bold.

**Table 3:** Summary of generalisation performance of the three classifiers trained with *O* = {5, 4, 3, 2} to new grasp poses (PT and RT datasets)

		Random Forest					
		Dense-Dataset		Baseline-4		Baseline-6	
Training Objects		PT	RT	PT	RT	PT	RT
**5**	Avr.	**0.40**	**0.51**	0.32	0.31	0.31	0.36
	std	0.04	0.07	0.06	0.07	0.09	0.14
**4**	Avr.	**0.42**	**0.54**	0.32	0.30	0.30	0.36
	std	0.07	0.10	0.09	0.14	0.10	0.15
**3**	Avr.	**0.38**	**0.57**	0.28	0.35	0.26	0.37
	std	0.08	0.14	0.12	0.10	0.14	0.14
**2**	Avr.	**0.34**	**0.47**	0.24	0.30	0.27	0.32
	std	0.14	0.28	0.17	0.23	0.17	0.25

		Support Vector Machine					
		Dense-Dataset		Baseline-4		Baseline-6	
Training Objects		PT	RT	PT	RT	PT	RT
**5**	Avr.	**0.52**	**0.48**	0.49	0.43	0.48	0.45
	std	0.08	0.08	0.07	0.06	0.07	0.09
**4**	Avr.	**0.53**	**0.53**	0.48	0.43	0.45	0.45
	std	0.06	0.09	0.09	0.11	0.09	0.09
**3**	Avr.	**0.49**	**0.51**	0.42	0.43	0.42	0.43
	std	0.12	0.13	0.14	0.11	0.14	0.13
**2**	Avr.	**0.43**	**0.45**	0.34	0.31	0.35	0.35
	std	0.17	0.24	0.15	0.18	0.16	0.19

		Multi Layer Perceptron					
		Dense-Dataset		Baseline-4		Baseline-6	
Training Objects		PT	RT	PT	RT	PT	RT
**5**	Avr.	**0.46**	**0.44**	0.27	0.29	0.33	0.41
	std	0.08	0.09	0.13	0.09	0.10	0.12
**4**	Avr.	**0.44**	**0.48**	0.24	0.26	0.30	0.38
	std	0.10	0.13	0.12	0.15	0.12	0.10
**3**	Avr.	**0.35**	**0.45**	0.15	0.21	0.23	0.35
	std	0.14	0.16	0.18	0.17	0.13	0.16
**2**	Avr.	**0.31**	**0.42**	0.15	0.19	0.14	0.24
	std	0.15	0.22	0.17	0.19	0.16	0.20

The average and standard deviation of MCCs are computed across three repetitions and all tested subsets for each data collection approach. The best results obtained for each model and number of objects used for training are highlighted in bold.

**Table 4:** Generalisation performance of the three classifiers on data corresponding to objects excluded from each training subset (with *O* = 5 objects)

	Random Forest						Support Vector Machine						Multi Layer Perceptron					
	Dense-Dataset		Baseline-4 (200 grasps)		Baseline-6 (300 grasps)		Dense-Dataset		Baseline-4 (200 grasps)		Baseline-6 (300 grasps)		Dense-Dataset		Baseline-4 (200 grasps)		Baseline-6 (300 grasps)	
Subset (Dense-Dataset # of grasps)	Avr.	std	Avr.	std	Avr.	std	Avr.	std	Avr.	std	Avr.	std	Avr.	std	Avr.	std	Avr.	std
**1** (164)	**0.26**	0.03	0.21	0.18	0.09	0.19	**0.42**	0.03	0.37	0.06	0.39	0.04	**0.48**	0.02	0.22	0.20	0.22	0.04
**2** (127)	**0.23**	0.02	0.14	0.02	0.12	0.19	0.37	0.08	0.41	0.03	**0.42**	0.03	**0.35**	0.03	0.21	0.15	0.20	0.03
**3** (126)	**0.36**	0.02	0.35	0.13	0.25	0.04	**0.63**	0.02	0.56	0.07	0.60	<0.005	**0.46**	0.08	0.29	0.15	0.20	0.02
**4** (163)	**0.59**	0.02	0.50	0.03	0.51	0.12	**0.61**	0.01	0.56	0.07	0.53	0.08	**0.57**	0.01	0.48	0.05	0.49	0.10
**5** (163)	**0.67**	<0.005	0.28	0.08	0.36	0.09	**0.70**	<0.005	0.66	0.03	0.66	0.02	**0.62**	<0.005	0.40	0.10	0.55	0.02

The average and standard deviation of MCCs are computed across three repetitions of each data collection approach. The best results obtained for each model and subset are highlighted in bold.

**Table 5:** Summary of generalisation performance of the three classifiers trained with *O* = {5, 4, 3, 2} to unknown objects

	Random Forest					
	Dense-Strategy		Baseline-4		Baseline-6	
Training Objects	Avr.	std	Avr.	std	Avr.	std
**5**	**0.43**	0.19	0.30	0.16	0.27	0.20
**4**	**0.43**	0.17	0.30	0.15	0.30	0.15
**3**	**0.41**	0.11	0.26	0.11	0.26	0.12
**2**	**0.37**	0.09	0.25	0.10	0.26	0.11

	Support Vector Machine					
	Dense-Strategy		Baseline-4		Baseline-6	
Training Objects	Avr.	std	Avr.	std	Avr.	std
**5**	**0.55**	0.14	0.52	0.12	0.52	0.11
**4**	**0.56**	0.07	0.48	0.07	0.52	0.06
**3**	**0.56**	0.11	0.45	0.12	0.47	0.09
**2**	**0.54**	0.09	0.39	0.09	0.42	0.08

	Multi Layer Perceptron					
	Dense-Strategy		Baseline-4		Baseline-6	
Training Objects	Avr.	std	Avr.	std	Avr.	std
**5**	**0.50**	0.11	0.33	0.16	0.34	0.17
**4**	**0.47**	0.09	0.25	0.12	0.34	0.11
**3**	**0.41**	0.11	0.17	0.17	0.24	0.12
**2**	**0.36**	0.10	0.19	0.12	0.20	0.16

The average and standard deviation of MCCs are computed across three repetitions and all tested subsets for each data collection approach. The best results obtained for each model and number of objects used for training are highlighted in bold.

#### Generalisation to new grasp poses

We start by presenting the classifiers generalisation results to new object grasp poses when considering *O* = 5. The detailed generalisation performance of the three classifiers to new grasp poses of known objects, for each training subset of 5 objects, is reported in Table 2. In order to extract 5-object training data from Baseline-4 and Baseline-6 datasets, a total of 200 and 300 grasp experiments were required, respectively. On the other hand, generating each 5-object training data set from the Dense-dataset required only 149 grasps, on average.

The results show that classifiers trained with data extracted from Baseline-6 datasets tend to show better generalisation results than when trained on Baseline-4 datasets. This was expected since, as seen in Section “Tactile Slip Detection”, incorporating more grasp poses leads to training data with higher variability. However, the best performance is obtained for classifiers trained with Dense-datasets, collected via the proposed protocol—again, the results are supported by the findings in Section “Tactile Slip Detection”. Note that this observation is true for all tested subsets of five objects for both the Random Forest and MLP classifiers. For the SVM, the Dense-dataset leads to better generalisation on three subsets and remains very close to the best performance, especially when considering the standard deviation associated with those experiments, which is lower for Dense-datasets. In other words, we can conclude from these experiments that the variability of tactile data generated by our data collection protocol leads to slip detectors that can better generalise to novel grasp poses of known objects, while also requiring fewer grasping experiments. In addition, we can observe that the standard deviations computed across three repetitions of each subset are overall higher for both Baseline datasets than for Dense-datasets. This is evidence that, for independent repetitions of the different data collection approaches, the specific grasping poses considered when collecting data with Baseline-4 and Baseline-6 have a higher impact on the performance of the classifiers than repeating our data collection protocol multiple times. In other words, the results suggest that our data collection protocol leads to ML models that are more repeatable and less experiment-dependent, regardless of the subset of objects for which new grasp poses are tested.

Next, we discuss the results obtained when considering a varying number of training objects, *O* = {5, 4, 3, 2}. Since the number of training/testing subsets increases when fewer objects are considered for training, and due to space constraints, in Table 3 we provide a summary of the generalisation performance of each classifier to new grasp poses, for each number of objects considered during training. The results are reported as the mean MCC and associated standard deviation computed across all training and testing subsets for each given number of objects used for training, including the three repetitions of each data collection approach.

The same observations made for *O* = 5 objects can be made regardless of the number of objects (and therefore combinations of properties) present in the training set. In fact, even when considering fewer training objects, classifiers trained with Dense-datasets show a higher and more consistent generalisation performance to new grasp poses (i.e. higher mean MCC and lower standard deviation) than those trained with datasets resulting from baseline approaches. It is also important to note that the difference between the average MCC scores obtained with Dense-datasets and Baseline datasets is more prominent the fewer objects are used for training (see Fig. 9a). We argue that such results show the benefits of our approach, especially for use cases in which training data is limited. Finally, computing the average of the generalisation results to new grasp poses (PT + RT datasets), across the different numbers of objects used for training, *O* = {5, 4, 3, 2}, we note that models trained using the Dense-dataset improve up to 90% and 41% (best results obtained for the MLP classifier) compared to the Baseline-4 and Baseline-6 datasets, respectively.

#### Generalisation to unknown objects

Next, we are interested in quantifying the generalisation power to detect slips on new objects. Once again, classifiers are trained with data obtained using the same three data collection processes. However, in this case, the testing sets consist of all the test data collected for all objects that are not part of the training subsets. This includes the experiments of the Parallel-Test and Rotated-Test. Table 4 reports the performance of the three classifiers when trained on each subset of *O* = 5 objects, for each dataset.

We can observe that when the classifiers are tested on data collected for unknown objects, classifiers trained with datasets related to the Baseline-4 and Baseline-6 approaches suffer from worse generalisation power than those trained with Dense-datasets. In fact, except for a single tested subset for which the SVM classifier shows the best performance with the Baseline-4 datasets, all three classifiers demonstrate their best classification performance on new objects when trained with the Dense-Datasets. It is also apparent that across repetitions of the same experiments, and for most subsets of 5 training objects, data collected with the DENSE protocol lead to slip classifiers exhibiting less variability in their respective performances, as supported by the lower standard deviation values obtained across tested subsets.

Similarly to the previous section, a summary of the generalisation power of each classifier to new objects computed across all subsets and repetitions for each *O* = {5, 4, 3, 2} is reported in Table 5. The conclusions drawn in the previous section (Table 3) also largely apply to the results presented in this table.

As expected, all classifiers do not perform equally for each number of object sets. For instance, for *O* = 5 objects, the overall MCC of the SVM (0.55) is larger than for both the RF (0.43) and ML (0.50). However, for each number of objects considered for training, classifiers fit on data collected using the DENSE protocol lead to better and more consistent generalisation results for new objects. This means that the variability contained in the dataset collected with the proposed approach further allows tactile slip detection models to better generalise to objects with different sets of properties (e.g. coefficient of friction or geometry) than models trained with typical data collection approaches reported in the literature^14,24,38^. Computing the average of the generalisation results to new grasps on new objects, across the different numbers of objects used for training, *O* = {5, 4, 3, 2}, we note that, models trained using the Dense-dataset improve up to 85% and 55% (best results obtained for the MLP classifier) compared to the Baseline-4 and Baseline-6 datasets, respectively.

## Discussion

In this work, we present a novel protocol for collecting tactile datasets containing slip events. The DENSE protocol is easier, faster, more efficient, and, most importantly, more reproducible than existing approaches^14,24,38,39^. This is achieved by systematic sampling of robotic grasp configurations based on the dimensions of each object (and sensor), thus making the DENSE protocol object and sensor-independent. The protocol is suitable for a broader set of grippers (i.e. any gripper that is able to close the fingers on an object, without requiring fine motor control); it requires fewer and simpler robotic actions (up to less than 50% grasps compared to previously proposed approaches) and a fast and easy labelling procedure (i.e. weak labelling); it produces data with a higher variability, thus better representing a wide range of gripper-object interactions that are expected of robotic grasping in real-world unstructured environments; and it permits to train slip detection models that show better generalisation to unseen objects and grasp poses, as proven by our experimental results using different machine learning models—up to 85% generalisation improvement to both new grasps (of the same objects) and new objects.

By analysing the MCC score, we show that classifiers trained with our Dense-dataset show a higher degree of correlation (between samples and predictions) than those trained with data collected using existing protocols, indicating better generalisation capabilities to new grasps and objects. While in this work we focus on the specific task of binary slip detection during pick and place, the general idea (i.e. structuring the data collection process to obtain the most diverse data with easy labelling, based on the experimenter’s understanding of the important aspects of the task) could be applied to data collection procedures for different tasks; for example, with a different choice of gripper poses, motions and grasping forces, the procedure outlined in Section “Methods” could generate diverse data for predicting incipient slip, modulating grip force, or reacting to external forces other than gravity. Moreover, although illustrated with a specific choice of tactile sensor and gripper, we believe that our approach is general and can be applied to a wide variety of sensors and robots. We, in fact, encourage the robotics community to make use of the code and dataset that we have made publicly available to test our data collection protocol with different robotic setups and to train new models with the Dense-dataset, to further extend our findings and to flag possible limitations.

## Methods

### Slip definition and tactile sensor

Slips are stick-slip phenomena characterised by sudden changes in the gripper-object state. These lead to unexpected variations of the object pose with respect to the gripper. Slips can be rotational or translational and will generally result in partial or complete loss of contact, which in turn weakens the shear forces acting on the fingers^47^.

Slips are often formulated as perturbations in the frictional system between a gripper and an object that can be described by the model of Coulomb:

$$\begin{array}{ll}{F}_{t}\le{\mu }_{s}{F}_{n},\quad {\rm{Static}}\,{\rm{friction}}\\{F}_{t}={\mu }_{k}{F}_{n}.\quad {\rm{Kinetic}}\,{\rm{friction}}\end{array}$$

where *μ*_*s*_ and *μ*_*k*_ are the static and kinetic coefficients of friction, respectively. This model implies that an object is held statically as long as the normal grip force *F*_*n*_ counteracts the tangential forces *F*_*t*_ applied to the contact surface. Therefore, slips occur when this balance is disturbed, and the held object starts experiencing kinetic friction.

**Fig. 10:**
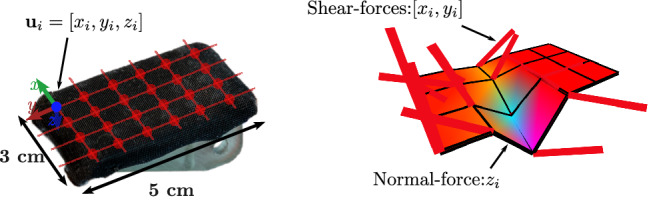
Left—Position of the 24 taxels composing the uSkin sensor. Each taxel *i* measures the local magnetic field, denoted as u_*i*_, which is non-linearly correlated to the normal and shear forces applied to the sensing unit. Right—uSkin tactile imprint. The normal components are represented by the surface deformation and colour (blue if the detected forces are high, red otherwise), while the local shear forces are depicted by the fixed-length, directional red line segments.

In this work, we detect slips with a tactile sensor based on magnetic technology^42^. In general, these sensors measure the deformation of a soft material as induced by external contacts (i.e. the tactile stimuli) by tracking the resulting movements of magnetic sources that are embedded in the soft material^48–50^. We use a specific version of the uSkin sensor^41,51^, which features 24 taxels (i.e. sensing units) distributed as a 6 × 4 matrix (see Fig. 10). Each taxel is made of a silicone dome embedding a magnet located on top of a 3D Hall effect sensor, updating its values at 100 Hz. Therefore, the raw data measured at each taxel, denoted as ${{\bf{u}}}_{i}\in {{\mathbb{Z}}}^{3}$, represents the values of the local 3D magnetic field induced by the position of the corresponding magnet.

The forces applied to the sensor induce both shear (*F*_*t*_) and normal (*F*_*n*_) forces across its surface. Hence, at each taxel *i* ∈ {1, …, 24}, we have the following:

$$\begin{array}{ll}{{\bf{u}}}_{i}=[{x}_{i},{y}_{i},{z}_{i}],\\{{\bf{u}}}_{i}=g({F}^{i}),\quad {F}^{i}={F}_{t}^{i}+{F}_{n}^{i},\end{array}$$

where ${F}^{i},{F}_{t}^{i}$ and ${F}_{n}^{i}$ correspond to the local net, shear and normal force applied to taxel *i*, and *g* is an unknown non-linear function. The values mostly correlated to the distributed shear forces are *x*_*i*_ and *y*_*i*_, whereas *z*_*i*_ mostly carry information related to normal forces. At a given time *t*, a full tactile sample will be denoted as a 24 × 3 matrix ${{\bf{U}}}_{t}={[{{\bf{u}}}_{1}^{t},...,{{\bf{u}}}_{24}^{t}]}^{T}$.

Given the robotic setup illustrated in Fig. 2, detecting slips is equivalent to determining if a tactile sample **U**_*t*_ corresponds to an instance of an unstable contact between a gripper and an object.

Slip detection can be formulated as a classification problem, which consists of learning an approximation of the function *f*: **U**_*t*_ → *Y*, where *Y* ∈ {0, 1}, with *Y* = 0 denoting a static interaction between an object and the sensor and *Y* = 1 a slip.

### ML models parameters

Training ML classifiers requires tuning a set of hyperparameters (e.g. number of neurons or decision trees, activation function, number of layers, learning rate, etc.) which play a crucial role in their respective performances. Although we explored multiple combinations of hyperparameters for each ML model, in this paper, we report only the performance of the overall best-performing ones, which correspond to:

**Random Forest (RF)**: consisting of 300 decision trees—each using a maximum of 25 features (i.e. maximum depth)—and using the Gini criterion to assess the quality of a data split.

**Support Vector Machine (SVM)**: featuring a Radial Basis Function as the model kernel function.

**Fig. 11:**
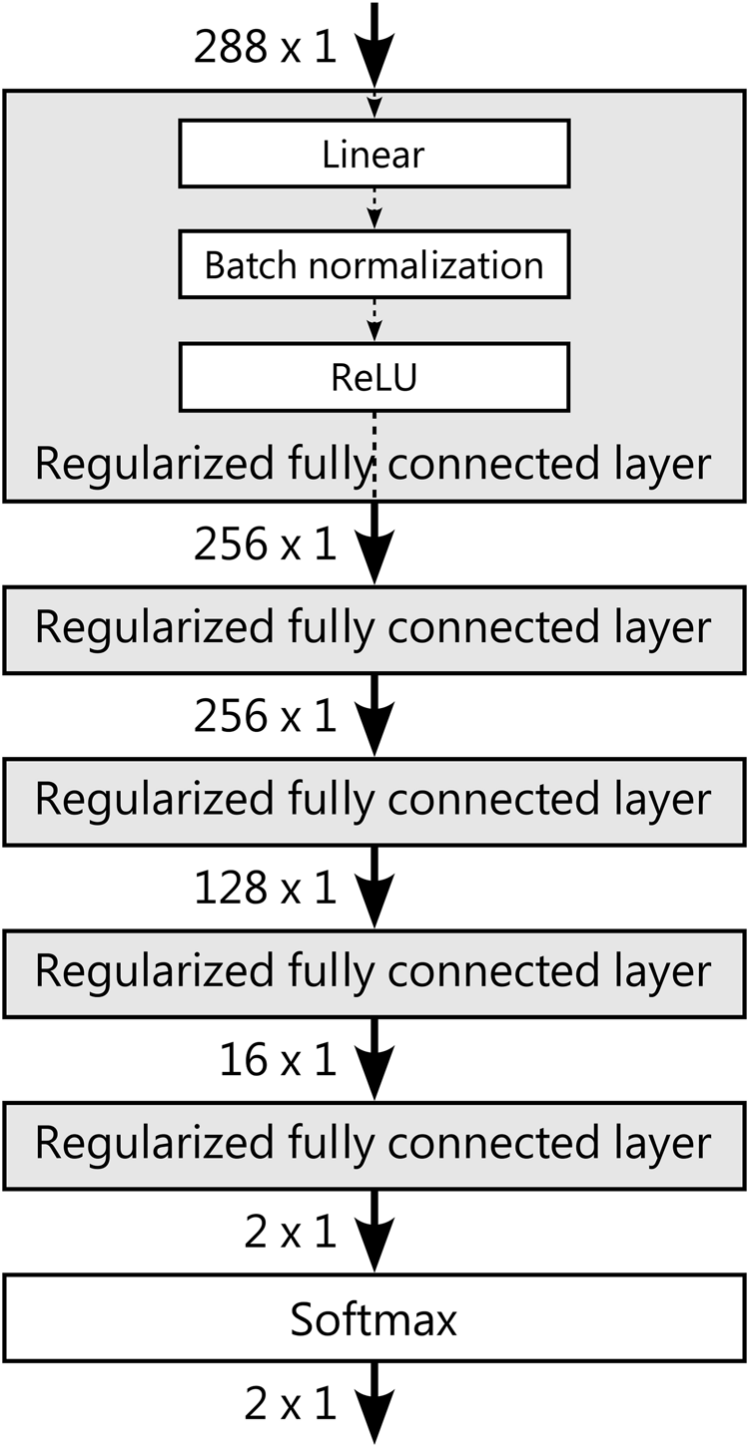
Architecture of the best-performing Multi Layer Perceptron model for which generalisation performance is reported in Section “Results”. A set of regularised, fully connected layers learn an abstract representation of input features that are then classified into ‘Slip’ and ‘No-Slip’ events.

**Multi-Layer Perceptron (MLP)**: following an architecture illustrated in Fig. 11. An Adam optimiser^52^ is used to minimise the Cross-Entropy Loss, over 100 epochs with an initial learning rate of 0.01 set to decrease via a Cosine Annealing scheduler.

#### Feature extraction

Since the data collected by the uSkin sensor is uncalibrated^41^, and that readings of the tactile sensor at rest can experience drifts after several experiments, we compute hand-crafted features to train classifiers:

$${\phi }_{t}=[{{\bf{U}}}_{t-n}-{{\bf{U}}}_{0},\cdots \,,{{\bf{U}}}_{t}-{{\bf{U}}}_{0}],\,t > n\ge 1.$$

In other words, for a sample captured at time *t* > *n* ≥ 1, we concatenate the previous *n* samples that are subtracted by the first reading of the corresponding experiment, i.e. after the object grasp and before the object lift. Training classifiers using these time windows accounts for the dynamic nature of slip events. However, *n* should be carefully selected to limit the size of the feature vector and avoid slowing down both training and prediction time. In our case, we selected *n* = 3, meaning that all classifiers are trained with features corresponding to 0.04 s.

### Matthews correlation coefficient

In this paper, we utilised the MCC to compare the performance of various classification models. Similarly to the F1-score^53^, the MCC evaluates the quality of binary classification models, but considers all four entries (true positive TP, true negative TN, false positive FP, and false negative samples FN) of confusion matrices:

$${\rm{MCC}}=\frac{({\rm{TP}}\times {\rm{TN}})-({\rm{FP}}\times {\rm{FN}})}{\sqrt{({\rm{TP}}+{\rm{FP}})({\rm{TP}}+{\rm{FN}})({\rm{TN}}+{\rm{FP}})({\rm{TN}}+{\rm{FN}})}},$$

Unlike the F1-score, which can attain high values despite low true negative predictions^46^, this metric provides information relative to the correlation between the predicted and the actual classes, where 1 indicates a perfect prediction, 0 indicates no better than a random prediction, and −1 indicates total disagreement between the predicted and the actual sample classes. In our previous work^11^, we noted that high F1-scores could still be obtained despite low TN values. In fact, the MCC has been recommended as a fairer and more reliable statistical metric for binary classification performance analysis^54^.

## Data Availability

All data generated and analysed in this paper are available from the corresponding author upon request, and are open-sourced at Github, https://github.com/ARQ-CRISP/slip_detection_dataset_2025.
